# A checklist of the vascular plants of the Democratic Republic of the Congo

**DOI:** 10.3897/phytokeys.277.193807

**Published:** 2026-06-29

**Authors:** Pierre Meerts, Petra Ballings, Patricia Barberá, Kenneth Bauters, Henk Beentje, Martin Cheek, Thomas L. P. Couvreur, Theo H. J. Damen, Rafael Felipe de Almeida, Petra De Block, Manuel de la Estrella, Steven Dessein, Henry Engledow, Eberhard Fischer, Laurent Gautier, Anna Haigh, David J. Harris, Wilbert Hetterscheid, Carel C. H. Jongkind, Olivier Lachenaud, Isabel Larridon, Alexandra Ley, Olivier Maurin, Simon J. Mayo, Pélagie Mbandu Luzolawo, Chanelle Moafo Tchioffo, Jeremie Morel, Ithe Mwanga Mwanga, Marco O. O. Pellegrini, Alejandro Quintanar, Marc Reynders, Elmar Robbrecht, Butoto Imani wa Rusaati, Ana R. G. Simões, Marc S. M. Sosef, Tariq Stévart, Robert D. Stone, Alexander P. Sukhorukov, Ton van der Zon, Brecht Verstraete, Justyna Wiland-Szymańska, Martin Xanthos, Fernando Zuloaga

**Affiliations:** 1 Meise Botanic Garden, Nieuwelaan 38, 1860 Meise, Belgium Ghent University Gent Belgium; 2 Université Libre de Bruxelles, Av. F.D. Roosevelt 50, CP 244, 1050 Brussels, Belgium Royal Botanic Gardens Kew London United Kingdom; 3 Fédération Wallonie-Bruxelles, Rue A. Lavallée 1, 1080 Brussels, Belgium Lomonosov Moscow State University Moscow Russia; 4 Missouri Botanical Garden, Saint Louis, MO, 63110-2291 USA Centro de Investigación en Biodiversidad y Cambio Global (CIBC-UAM), Universidad Autónoma de Madrid Madrid Spain; 5 Biology Department, Universidad Autónoma de Madrid, Madrid, 28049 Spain Biology Department, Universidad Autónoma de Madrid Madrid Spain; 6 Centro de Investigación en Biodiversidad y Cambio Global (CIBC-UAM), Universidad Autónoma de Madrid, Madrid 28049, Spain Meise Botanic Garden Meise Belgium; 7 Ghent University, K.L. Ledeganckstraat 35, 9000 Gent, Belgium Université Libre de Bruxelles Brussels Belgium; 8 Royal Botanic Gardens Kew, Richmond, TW9 3AE, London, United Kingdom Royal Botanic Garden Edinburgh Edinburgh United Kingdom; 9 DIADE, Univ Montpellier, CIRAD, IRD, Place E. Bataillon, 34095 Montpellier Cedex 5, Montpellier, France Herbarium MA, Real Jardín Botánico-CSIC Madrid Spain; 10 Naturalis Biodiversity Centre, Botany Section, Darwinweg 2, 2333 CR Leiden, Netherlands Jardin Botanique de Genève Chambésy Switzerland; 11 Wageningen, Netherlands University of the Witwatersrand Johannesburg South Africa; 12 University of the Witwatersrand, Johannesburg, 2092, South Africa Institut de Systématique, Évolution, et Biodiversité (ISYEB), Unité Mixte de Recherche 7205, Muséum National d’Histoire Naturelle, CNRS, Sorbonne Université, EPHE-PSL, Université des Antilles Paris France; 13 Universidad de Córdoba, Av. Medina Azahara 5, 14015 Córdoba, Spain Institut für Integrierte Naturwissenschaften-Biologie, Universität Koblenz Koblenz Germany; 14 Institut für Integrierte Naturwissenschaften-Biologie, Universität Koblenz, Universitätsstraße 1, 56070 Koblenz, Germany Adam Mickiewicz University Poznań Poland; 15 Jardin Botanique de Genève, ch. de l’Impératrice 1, C.P. 71, 1292 Chambésy, Switzerland Fédération Wallonie-Bruxelles Brussels Belgium; 16 Royal Botanic Garden Edinburgh, 20A Inverleith Row, Edinburgh EH3 5LR, United Kingdom University of KwaZulu-Natal Pietermaritzburg South Africa; 17 Institut de Systématique, Évolution, et Biodiversité (ISYEB), Unité Mixte de Recherche 7205, Muséum National d’Histoire Naturelle, CNRS, Sorbonne Université, EPHE-PSL, Université des Antilles, CP 39, 57 rue Cuvier, F-75005 Paris, France Missouri Botanical Garden Saint Louis United States of America; 18 Martin Luther University Halle-Wittenberg, Universitätsplatz 2, 06108 Halle (Saale), Germany DIADE, Univ Montpellier Montpellier France; 19 Institut Supérieur Pédagogique de la Gombe, Kinshasa, Democratic Republic of the Congo Naturalis Biodiversity Centre, Botany Section Leiden Netherlands; 20 Centre de Recherche en Sciences Naturelles, Lwiro, Sud-Kivu, Democratic Republic of the Congo Martin Luther University Halle-Wittenberg Halle Germany; 21 Herbarium MA, Real Jardín Botánico-CSIC, Plaza de Murillo, 2, 28014 Madrid, Spain Universidad de Córdoba Córdoba Spain; 22 Sangju City, Republic of Korea Unaffiliated Wageningen Netherlands; 23 University of KwaZulu-Natal, Pietermaritzburg 3209, South Africa Institut Supérieur Pédagogique de la Gombe Kinshasa Democratic Republic of the Congo; 24 Lomonosov Moscow State University, Leninskiye Gory, Moscow 119991, Russia Centre de Recherche en Sciences Naturelles Lwiro Democratic Republic of the Congo; 25 Adam Mickiewicz University, ul. Uniwersytetu Poznańskiego 6, 61-614 Poznań, Poland Unaffiliated Sangju City Republic of Korea; 26 Darwinion Institute of Botany, Labarden 200, 1642, San Isidro, Buenos Aires, Argentina Darwinion Institute of Botany Buenos Aires Argentina

**Keywords:** Biodiversity conservation, botanical collections, Central Africa, endemic, herbarium specimens

## Abstract

A checklist of the vascular flora of the Democratic Republic of the Congo (DRC) has been assembled, based on literature, online databases, and herbarium collections. It includes 10,260 species (38 lycophytes, 347 monilophytes, 16 gymnosperms, and 9,859 angiosperms), belonging to 246 families. The five largest families are Fabaceae (11.8% of specific richness), followed by Rubiaceae (6.7%), Asteraceae (6.6%), Poaceae (6.1%), and Orchidaceae (5.6%). Strict endemics of the DRC amount to 1,099 species, i.e., 10.7%. Families represented by > 120 species and comprising a high proportion of endemics include Commelinaceae (endemism rate: 24%), Euphorbiaceae (21%), Asteraceae (16%), and Fabaceae (16%). A total of 461 introduced species are recorded outside cultivation in the country, i.e., 4.5% of the total flora. Solanaceae, Brassicaceae, and Amaranthaceae stand out with their higher-than-average proportions of introduced species. Herbs represent the most frequent life form (54%), followed by (sub)shrubs (17%), trees (14.5%), climbers (10%), and epiphytes (4%). The IUCN conservation status was retrieved for 2,633 species (25.7%), of which two species are extinct and 448 (17%) are assigned to a threat category (CR [Critically Endangered]: 36; EN [Endangered]: 184; VU [Vulnerable]: 228). IUCN status was retrieved for 184 (17%) of the endemic species, of which 135 (73%) are considered extinct or threatened.

## Introduction

The erosion of biodiversity, particularly in tropical regions, represents a major challenge for the future of humanity (Dirzo et al. 2014; IPBES 2019). Under the Convention on Biological Diversity, countries are responsible for the conservation of biological diversity within their territories, in particular their endemic species (CBD 1992). In this context, reliable biodiversity inventories are essential for informed decision-making and effective conservation action (Groombridge and Jenkins 2002; Kühl et al. 2024). Paradoxically, such inventories remain highly incomplete in many tropical regions, despite the fact that these regions harbor most of the world’s biodiversity (Gaston and Williams 1996; Raven et al. 2020). Access to reliable and comprehensive information on the status of biodiversity is therefore indispensable for the development, implementation, and evaluation of conservation policies (IPBES 2019).

In tropical Africa, considerable efforts have been made to produce country-level checklists of the vascular plant flora (Angola: Figueiredo and Smith 2008; Benin: Souza 2009; Botswana: Setshogo 2005; Burkina Faso: Lebrun et al. 1991; Zizka et al. 2015; Burundi: Ntore et al. 2024; Cameroon: Onana 2011; Chad: Brundu and Camarda 2013; Equatorial Guinea: Velayos et al. 2013, 2014, 2023; Gabon: Sosef et al. 2006; Guinea: Gosline et al. 2023; Guinea-Bissau: Catarino et al. 2008; Ivory Coast: Aké-Assi 2001, 2002; Mali: Boudet et al. 1986; Mozambique: Odorico et al. 2022; Niger: Peyre de Fabrègues and Lebrun 1976; São Tomé and Príncipe: Figueiredo et al. 2011; Senegal: Lebrun 1973; Zambia: Phiri 2005), notably driven by the Global Strategy for Plant Conservation. The production of these inventories is now greatly facilitated by the availability of online resources, including digitized specimen data and taxonomic backbone information within global facilities such as the World Flora Online (Borsch et al. 2020) and the World Checklist of Vascular Plants (WCVP, https://powo.science.kew.org/).

The Democratic Republic of the Congo (DRC) is the second-largest country in Africa. Located at the heart of Central Africa, with the second-largest continuous rainforest surface in the world, it is one of the regions with exceptionally rich biodiversity and a major stake in the fight against climate change (de Wasseige et al. 2012). Even though the country’s flora is covered by the Flore d’Afrique centrale series (1948–; see below) (Sosef 2016), which is now 70% complete, it still lacks an up-to-date, comprehensive, and taxonomically reliable inventory of its vascular plant flora. The “National Biodiversity Strategy and Action Plan of D.R. Congo” (Anonymous 2016) highlights the lack of taxonomic knowledge of the flora and the urgent need for updated inventories. The present work aims to fill this gap and address this issue. Particular attention is paid to endemic species, for which DRC has an obvious conservation responsibility.

### The study region

DRC is highly diverse in terms of climate, topography, and natural formations. The central part of the country is occupied by a vast sedimentary basin, the Congo River Basin, which is generally at an altitude below 500 m (Robert 1942). The climate is equatorial (Af and Am types in the Köppen classification (Peel et al. 2007)). Soils are mostly ferralsols (Ngongo et al. 2009). The dominant vegetation is dense, humid forest. It is made up of a variety of forest types, some of which are swampy or periodically flooded (Lebrun and Gilbert 1954). This region belongs to the Guineo-Congolian phytoregion (White 1983), stretching from Guinea to the Republic of the Congo and northern Angola, and includes most of the DRC.

The Congo Basin is surrounded, except to the west, by plateaus and mountains that rise progressively. To the north and south of this equatorial belt, the climate becomes tropical (Aw type), characterized by increasing seasonality. The forest gradually gives way to various types of tropophilous formations, ranging from woodlands to grasslands. The extreme north of the country belongs to the Guineo-Congolian/Sudanian transition zone (White 1983).

Regions located south of the Guineo-Congolian region largely belong to the Guineo-Congolian/Zambezian transition zone (White 1983). The southeastern part of the country, corresponding to Haut-Katanga as defined by Robyns (1948a), belongs to the Zambezian domain. Altitudes range from approximately 600 m to approximately 2,400 m (Marungu Massif). The climate is of the Cwa and Cwb types and is characterized by a long dry season. The most widespread vegetation today is tropophilous woodland (miombo), but the main climax vegetation was probably dry evergreen forest (Schmitz 1963). The high plateaus and the flood-prone soils of western Katanga are occupied by savannas. Locally, the presence of copper- and cobalt-rich geological substrates gives rise to unique metalliferous soils that harbor a highly specialized endemic flora (Duvigneaud and Denaeyer-De Smet 1963; Faucon et al. 2010).

The eastern margin of DRC (Kivu and Ituri, in part) is part of the Afromontane phytoregion (White 1983). The climate is Cw. This region corresponds to the Albertine Rift, an active tectonic zone that has given rise to a series of mountains, volcanoes, and large lakes. Mount Stanley, in the Ruwenzori Massif, rises to over 5,100 m. The climate there is markedly cooler due to the altitude. The vegetation is complex and comprises diverse types of evergreen Afromontane forests and shrub formations dominated by Ericaceae. The highest peaks are occupied by a very distinctive Afroalpine vegetation (Robyns 1948b). The Albertine Rift, extending to Uganda, Rwanda, and Burundi, hosts many endemic species (Plumptre et al. 2007).

Several studies, based on statistical analyses of the vascular flora, have highlighted the high species richness and endemism of specific regions within the DRC, in particular the southeast (Haut-Katanga in the phytogeographic sense as defined by Robyns (1948a)) and east (Kivu and Ituri) (Linder 2001, 2014; Marshall et al. 2016; Sosef et al. 2017). These territories are recognized as “bioquality hotspots” for the tropical African flora, i.e., areas distinguished by a high proportion of rare species (Marshall et al. 2016).

DRC is experiencing rapid deforestation, mostly driven by the clearing of vegetation for agriculture, charcoal production, and mining (Mangaza Nondo et al. 2026; Shapiro et al. 2021), which is aggravated by political instability and protracted conflicts (Kelly et al. 2022). Currently, 21 species of plants are legally protected (Anonymous 2018). Moreover, the country has established a network of ca. 60 protected areas, covering approximately 312,139 km^2^, representing 13.3% of the national territory. There are seven national parks, four of which are listed as UNESCO World Heritage Sites. Nevertheless, the management of national parks faces serious challenges due to deforestation, growing anthropogenic pressure, armed conflicts, insufficient human and financial resources, and governance shortcomings (Anonymous 2016).

### Exploration and research on plant biodiversity in the Democratic Republic of the Congo

Botanical exploration in the DRC began with the expedition of J. H. Tuckey in 1816, during which Christen Smith made the first botanical collections (now in BM). However, a long interlude followed before intensive collecting resumed under Belgian colonization. Therefore, vascular plants collected in the DRC before the 20^th^ century account for approximately 8,000 specimens in the Meise Botanic Garden collections (BR), i.e., less than 2% of the total. Early collections during the colonial period initially supported the research activities of the State Botanical Garden in Brussels (Belgium), which is now known as Meise Botanic Garden. Under the impetus of Émile De Wildeman, intense research activity into the taxonomy of Central African flora rapidly developed there. Botanical exploration expanded significantly with the establishment of the Institut National pour l’Étude Agronomique du Congo (INEAC), the headquarters of which were located in the central Congo Basin at Yangambi. The largest herbarium collection in the DRC is still housed there today (YBI). The most active period for plant collecting in the DRC occurred between 1930 and 1960 (Sosef et al. 2017), while specimens collected after 2000 represent ca. 5% of the total. The largest specimen collection for the DRC is currently held at Meise Botanic Garden (BR). Other important collections are hosted at BRLU (Paul Duvigneaud’s collections) and POZG (Stanisław Lisowski’s collections). Many other herbaria, notably BM, G, GENT, K, MO, P, and WAG, also hold significant collections, although these often consist of specimens for which a duplicate is also available at BR. The Index Herbariorum 2012 (https://sweetgum.nybg.org/science/ih) lists 13 herbaria in the DRC, but only a few of these remain active. Parts of the collections of some of these herbaria (e.g., KIN, LWI, LSHI, YBI) have been digitized with the support of Meise Botanic Garden (and funding from the Mellon Foundation) and are accessible via the JSTOR Global Plants portal (https://plants.jstor.org/). The total number of vascular plant herbarium records for DRC at BR is close to 500,000, corresponding to ca. 400,000 collecting events, i.e., ca. 0.17 gatherings/km^2^, much less than the recommended minimum number of one gathering/km^2^ (Campbell and Hammond 1989). Even though the number of additional gatherings in other herbaria is not known, it is clear that DRC is severely under-sampled. The 10 most prolific collectors of the DRC flora (Table 1) account for ca. one-third of all specimens collected to date.

**Table 1.** The 10 most prolific collectors of the flora of DRC.

	**Period of collecting activity in DRC**	**# specimens**	**Herbarium**
Joseph Bequaert	1913–1914	12,421	BR
Gaston-Francois de Witte (& coll.)	1932–1956	12,622	BR
Paul Duvigneaud	1948–1960	>50,000	BRLU
Jean Lebrun	1928–1957	16,431	BR
Stanisław Lisowski (& coll.)	1968–1981	36,490	BR, POZG
Jean Louis	1935–1940	29,219	BR
François Malaisse (& coll.)	1960–2008	ca. 15,000	BR
Paul Quarré	1920–1945	11,467	BR
Jean-Jacques Symoens (& coll.)	1952–1970	14,792	BR
Hyacinthe Vanderyst	1906–1933	40,753	BR

Publication of the Flore du Congo belge et du Ruanda-Urundi began in 1948 and is still ongoing today under the title Flore d’Afrique centrale (Sosef 2016). By 2010, it covered ca. 6,100 species of vascular plants found in the DRC, Rwanda, and Burundi. Since then, treatments corresponding to 45 new families, comprising 1,525 additional species, have been published. The complete text of the fascicles published up to and including 2023 is available online (https://www.plantentuinmeise.be/nl/flore-d-afrique-centrale-6z3b). Partial or complete floras and catalogs have been published for limited parts of the country, including Albert National Park (now Virunga National Park) (Robyns 1947, 1948b; Robyns and Tournay 1955), Garamba National Park (Troupin 1956), the Tshopo Basin (Lejoly et al. 2010), the trees and shrubs of Haut-Katanga (Meerts 2016; Meerts and Hasson 2016), rainforest trees (Doucet et al. 2025), the flora of the copper-cobalt outcrops of Haut-Katanga (Malaisse et al. 2016), and useful plants (Latham et al. 2025). Reliable data on endemism are available only for particular groups, such as trees (Sosef et al. 2021) and the specialized flora of copper-cobalt outcrops (Faucon et al. 2010).

DRC is currently one of the least-explored territories in Africa in terms of its flora (Küper et al. 2006; Stropp et al. 2016; Sosef et al. 2017). The degree of exploration varies greatly, with the number of gatherings being inversely related to the distance from cities (Koffi et al. 2008). The Albertine Rift mountains in the east and the areas surrounding major cities such as Lubumbashi, Kisangani, and Kinshasa are far better documented than the rest of the country. New species of vascular plants are regularly described based on material collected in the DRC.

## Methods

The aim is to report all the vascular plant species currently known to occur naturally in the DRC and their threat category for those that have been assessed so far. Infraspecific taxa and hybrids are excluded. A preliminary list of taxon names was compiled through an extensive review of different sources. Data were extracted from Plants of the World Online (http://www.plantsoftheworldonline.org), the African Plant Database (APD version 4.0.0, Conservatoire et Jardin botaniques de la Ville de Genève and South African National Biodiversity Institute, Pretoria, http://africanplantdatabase.ch), and the online databases of various herbarium collections, including Naturalis Biodiversity Centre (https://bioportal.naturalis.nl), the Botanical Collections resource hosted by Meise Botanic Garden (https://www.botanicalcollections.be/), and the AMUNATCOLL database of the Natural History Collections, Faculty of Biology of Adam Mickiewicz University in Poznań (https://amunatcoll.pl). Additional names were retrieved from the Flore d’Afrique centrale (https://www.plantentuinmeise.be/nl/flore-d-afrique-centrale-6z3b) and from data mining in recent floristic and taxonomic studies.

This draft list was shared with taxonomic and floristic specialists (all co-authors of the checklist) for validation. This included nomenclatural validation based on recent literature and confirmation of occurrence in the DRC. For almost all species, occurrence in the DRC is confirmed by direct or indirect reference to a herbarium specimen. The Flore d’Afrique centrale cites representative specimens for all species treated. Therefore, additional vouchers are not provided for the species included in the volumes of the Flore, provided that the taxonomic concept of the species has not changed since the Flore’s publication. For families not yet covered by the Flore, or those that are only partially covered, and for species that have been newly described or recorded in the DRC, experts were given some freedom regarding the nature of acceptable evidence that could be used to confirm the species’ presence in the DRC. For most species, only one voucher specimen is cited. These were retrieved from the Botanical Collections resource hosted by Meise Botanic Garden (https://www.botanicalcollections.be) and from GBIF and RAINBIO (Dauby et al. 2016) for other herbaria, in particular BRLU, K, MO, P, POZG, and WAG. However, assembling an exhaustive list of herbarium specimens was beyond the scope of the project. When selecting vouchers, preference was given to specimens identified by plant family specialists whenever possible. When there were duplicates of the same gathering with different identifications in different collections, the duplicate with the most recent identification label or made by a specialist for the relevant group was selected. The voucher information usually comprises the collector(s)’ name(s) and gathering number and, where available, a unique ID for that voucher, referring to the botanical institution where it is housed (usually a barcode). Alternatively, or in addition, a recent literature reference citing specimens from DRC is provided. In addition to the Flore d’Afrique centrale, accepted references include taxonomic monographs, revisions, phylogenetic studies, and nomenclatural updates when these include a full citation of the voucher specimen(s) collected in the DRC.

### Synonyms

The nomenclatural backbone of the checklist is that of the World Flora Online (https://www.worldfloraonline.org/), with only a few exceptions. Any taxon name accepted by the Flore d’Afrique centrale that is no longer considered an accepted name is included in the corresponding synonymy. Other synonyms are included only for recent nomenclatural changes, when Kew’s Plants of the World Online (POWO) does not recognize the same name as WFO or when standard floras such as Flora Zambesiaca, Flora of Tropical East Africa, and the Flore du Gabon use a different name. For recent nomenclatural changes not yet incorporated into databases, literature references are cited.

### Native and introduced species

Introduced species are included only if their occurrence outside cultivation (i.e., as casual, escaped from cultivation, naturalized, or invasive species) is confirmed by a voucher specimen, following Bordbar and Meerts (2022) most often. In a few cases, this reference is not followed, accepting the following species as native because they are generally considered so in neighboring countries and mostly occur in natural vegetation: *Chenopodiastrum fasciculosum* (Aellen) Mosyakin, *Cuscuta abyssinica* A.Rich., *Hilleria latifolia* (Lam.) H.Walter, *Hydrocotyle bonariensis* Comm. ex Lam., *H. ranunculoides* L.f., *Sida cordifolia* L., and *Solanum lichtensteinii* Willd.

### Endemic taxa

Taxa from DRC that lacked verified records from any other country were classified as endemic. The near-endemic category was not considered.

### Life form

Each species was assigned to one of the following six life forms: tree, shrub or subshrub, herb, climber (herbaceous or woody), epiphyte, or epiphytic parasite. The information was retrieved from the Flore d’Afrique centrale, POWO, or expert advice. Shrubs and subshrubs were pooled because the distinction is not easily defined or consistently applied to all families. Somewhat arbitrary decisions had to be made when intermediate and doubtful cases were encountered.

### Conservation status

IUCN Red List categories were retrieved from the IUCN website (https://www.iucnredlist.org/).

## Results and discussion

### Number of species

A total of 10,260 species, 1,998 genera, and 246 families of vascular plants have been recorded in the DRC (Suppl. material 1). DRC comprises 22% of the species richness of sub-Saharan Africa (based on data in Gautier et al. 2012). Of these, 96% are angiosperms (Table 2). Gymnosperms are very poorly represented, which is a typical pattern for tropical Africa in general (Mutke and Barthlott 2005). Pteridophytes account for less than 4% of the vascular flora, which is similar to their contribution to the vascular flora worldwide (Qian et al. 2022).

**Table 2.** Taxonomic assemblage of the vascular flora of DRC and contribution of endemics and introduced species. The Dicotyledonae represent the informal groups ANA + Magnoliids + Ceratophyllales + Eudicots; the Monocotyledonae equal the informal group Monocots; see APG IV (2016) for further explanation.

		**# native non endemics**	**# endemics**	**# introduced**	**Total**
Angiosperms					
	Dicotyledonae	6260	890	395	7545
	Monocotyledonae	2054	197	63	2314
Gymnosperms					
	Cycadophytes	6	1	0	7
	Gnetophytes	3	0	0	3
	Pinophytes	6	0	0	6
Pteridophytes					
	Lycophytes	32	6	0	38
	Monilophytes	339	5	3	347
Total		8700	1099	461	10260

**Figure 1.**
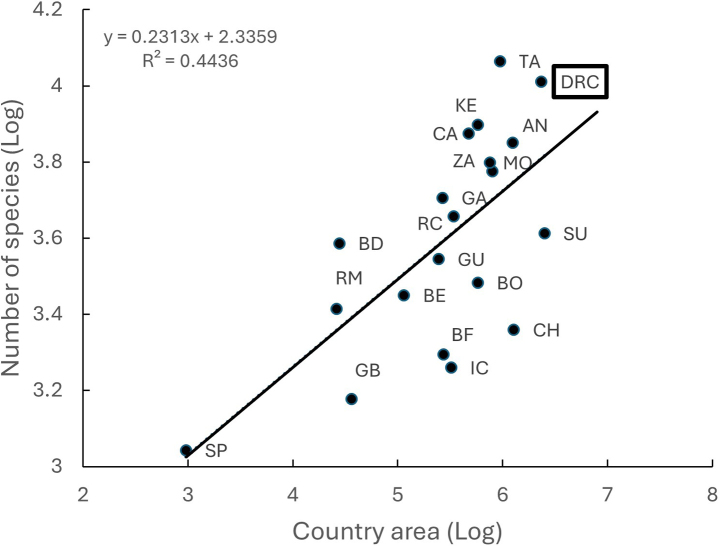
Correlation between species richness of vascular plants and country surface area in 20 tropical African countries and regions. AN Angola, BE Benin, BR Burundi, BO Botswana, BU Burkina Faso, CA Cameroon, CH Chad, DRC D.R. Congo (framed), GA Gabon, GB Guinea-Bissau, GU Guinea, IC Ivory Coast, KE Kenya, MO Mozambique, RC Republic of the Congo, RM Rio Muni, SP São Tomé and Príncipe, SU Sudan and South Sudan, TA Tanzania, ZA Zambia. See Table 3 for the sources of information. The equation is the linear regression line.

With 10,260 species, the DRC has one of the highest levels of floristic richness among tropical mainland African countries, surpassed only by Tanzania (Table 3 and references therein). This is not surprising given that DRC is the largest country in tropical Africa. As a fundamental ecological pattern, species number increases with sampling area, and the species-area relationship (SAR) follows a power law (*S* = *cA^z^*, where *S* is species richness and *A* is sampling area) (MacArthur and Wilson 1967). This relationship is generally expressed as a straight line on a log-log scale. Using the equation of the line across 20 tropical African countries (Fig. 1), based on DRC’s surface area, the expected floristic richness is 6,446, i.e., considerably less than the observed richness. Such a relatively high richness is consistent with the country’s high phytogeographic and ecological diversity. Three major drivers of the floristic assemblage in tropical Africa, i.e., annual rainfall, mean annual temperature, and altitude (Marshall et al. 2021), are highly variable in the DRC. The country spans four major “phytochoria” as defined by White (1983) (Sudanian, Zambezian, Guineo-Congolian, and Afromontane) and four floristic regions as defined by Droissart et al. (2018). Its flora comprises five distinct species groups as defined by Linder (2014): (i) lowland forest flora, which is predominantly represented in the Guineo-Congolian region; (ii) savanna flora, which occurs mainly in northern, southern, and eastern DRC; (iii) tropical montane flora, which is found mostly in the Albertine Rift; (iv) tropical alpine flora, which is restricted to the Ruwenzori Massif; and (v) a minor contribution from the austrotemperate flora, which is locally present in Kivu and Katanga. The only major sub-Saharan African floristic element absent from DRC is the flora of arid regions.

**Table 3.** Floristic richness, endemism, and introduced species in 20 tropical African countries and regions. * indicates that infraspecific taxa were included. References: (1) Goyder and Gonçalves 2019; Heinze et al. 2017; (2) Souza 2009; (3) Setshogo 2005; (4) Zizka et al. 2015; (5) Ntore et al. 2024; Meerts et al. (unpubl.); (6) Onana 2011; (7) Brundu and Camarda 2013; (8) Nicolas Texier (pers. comm.); (9) Gosline et al. 2023; (10) Catarino et al. 2008; (11) Aké-Assi 2001, 2002; (12) Quentin Luke (pers. comm.); (13) Odorico et al. 2022; (14) Lachenaud 2009; (15) Velayos et al. 2023; (16) Figueiredo et al. 2011; (17) Darbyshire et al. 2015; Omer et al. 2021; (18) Quentin Luke (pers. comm.); (19) Phiri 2005.

**Country**	**area (km^2^)**	**# species**	**# endemics**	**% endemics**	**# introduced**	**% introduced**
Angola (1)	1,246,700	7080	997	14.1	226	3.2
Benin (2)	114,763	2807	4	<0.1%	--	--
Botswana (3)	581,730	3041*	15	0.5	--	--
Burkina Faso (4)	274,000	1972	1	<0.1	116	5.9
Burundi (5)	27,830	3860	135?	3.5	206	5.3
Cameroon (6)	475,442	7501	500–585	6.7–7.8	185	2.5
Chad (7)	1,280,000	2288	55	2.4	274	12.0
DRC (this study)	2,345,000	10260	1099	10.7	461	4.5
Gabon (8)	267,667	5082	406	8	198	3.4
Guinea (9)	245,857	3505	81	2.3	396	11.3
Guinea Bissau (10)	36,125	1507*	3	0.2	48	3.2
Ivory Coast (11)	322,460	3853	73	1.9	--	--
Kenya (12)	582,646	7896	665	8.4	651	8.2
Mozambique (13)	801,590	5957	278	4.7	602	10.1
Republic of the Congo (14)	342,000	4538	15	0.3	--	--
Rio Muni (Equatorial Guinea) (15)	26,017	2598	11	0.4	152	5.8
São Tomé and Príncipe (16)	964	1104	107	9.7	301	27.3
Sudan and South Sudan (17)	2,505,825	4096	69	1.7	113	2.8
Tanzania (18)	947,300	11604	2200	19	495	4.3
Zambia (19)	753,000	6280*	212	3.4	141	2.2

New species are being described continuously based on specimens collected in the DRC. Since 2010, the International Plant Names Index (IPNI) (https://www.ipni.org/) has published 183 new names with types from DRC (data on 15 May 2026). New records are also regularly being added to the flora of DRC. For example, a recent revision of the genus *Coleus* Lour. (Lamiaceae) revealed that 14 of the 86 species present in the country were new records, and 15 species were new to science (Meerts and Paton 2024). Existing collections still comprise a substantial number of unidentified specimens, particularly those belonging to families not yet covered by the Flore d’Afrique centrale. It is certain that the number of vascular plant species recorded in the DRC will continue to rise in the coming years.

### Taxonomic spectrum

**Figure 2.**
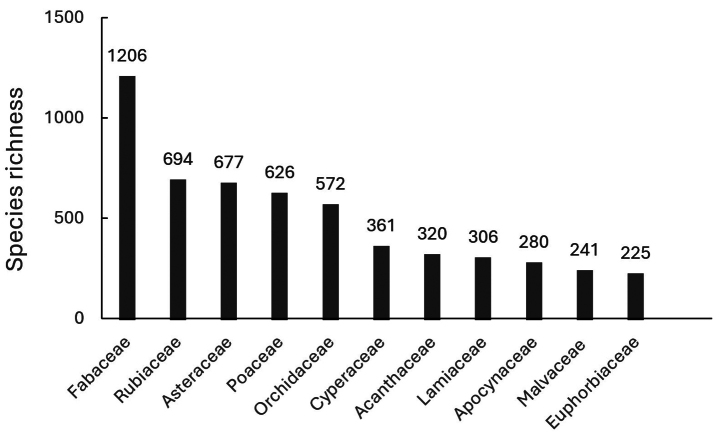
Number of species in the 10 largest families in the vascular flora of DRC.

A total of 246 vascular plant families are represented in the DRC, accounting for ca. 83% of those found in sub-Saharan Africa (Suppl. material 1). The 20 largest families, comprising 95 species or more, account for 64% of the total flora, while 49 families (20%) are represented by just one species. The largest families (> 500 species) include Fabaceae Lindl., which is far ahead of Rubiaceae Juss., Asteraceae Bercht. & J.Presl, Poaceae Barnhart, and Orchidaceae Juss. (Fig. 2). This is the same pattern as observed in tropical Africa in general (Klopper et al. 2007; Gautier et al. 2012). However, Rubiaceae, Orchidaceae, and Cyperaceae are overrepresented in the DRC (≥ 30% of the total species number in sub-Saharan Africa), while Euphorbiaceae and Asteraceae are underrepresented (17% of the total species number in sub-Saharan Africa). Concerning Euphorbiaceae, this may be due to the fact that major parts of the family, including *Euphorbia* L. and *Acalypha* L., have not yet been revised for DRC, and many specimens in collections remain unidentified.

The largest genera (Table 4) are essentially the same as those found in tropical Africa as a whole (Klopper et al. 2007; Gautier et al. 2012). Discrepancies reflect the use of different taxonomic concepts (e.g., *Cyperus* L., which has recently been expanded considerably (Larridon et al. 2014)). *Crotalaria* L. is remarkably overrepresented in the DRC, with ca. 40% of the number of species in sub-Saharan Africa.

**Table 4.** The 20 largest genera in the vascular flora of DRC.

**Genus**	**# species**
*Crotalaria* L.	197
*Cyperus* L.	162
*Vernonia* Schreb. (s.l.)	125
*Habenaria* Willd.	99
*Coleus* Lour.	86
*Indigofera* L.	84
*Polystachya* Hook.	77
*Combretum* Loefl.	74
*Psychotria* L.	74
*Ipomoea* L.	67
*Euphorbia* L.	64
*Eulophia* R.Br. ex Lindl.	64
*Commelina* L.	62
*Ficus* L.	60
*Asplenium* L.	59
*Helichrysum* Mill.	58
*Justicia* L.	55
*Phyllanthus* L.	55
*Bulbostylis* Kunth	53
*Cyphostemma* (Planch.) Alston	51

### Endemism

With 1,099 strict endemic species (Suppl. material 1), DRC hosts the second-highest number of endemic species among the tropical mainland African countries listed in Table 3, after Tanzania (Table 3). The rate of species endemism (10.7%) is surpassed only by Tanzania (19%) and Angola (14%) (Table 3). Globally, the number of endemic species (*E*) in a territory increases exponentially with area (*A*) (*E* = 0,00001227 · *A*^1.195^) (Hobohm et al. 2019). Based on the country’s area, the expected number of endemics is 502, less than half of the observed number.

Seven genera are endemic to the DRC, i.e., *Bampsia* Lisowski & Mielcarek (Linderniaceae), *Ewangoa* O.Lachenaud & Barberá (Euphorbiaceae), *Karina* Boutique (Gentianaceae), *Lebrunia* Staner (Clusiaceae), *Michelsonia* Hauman (Fabaceae), *Nelmesia* Van der Veken (Cyperaceae), and *Schaueriopsis* Champl. & I.Darbysh. (Acanthaceae). No endemic family was recorded.

Endemism in the DRC is very unevenly distributed geographically, with concentrations near the eastern and southeastern borders of the country (Kivu and Katanga) (Ndjele 1988; Mandango and Ndjele 1994; Sosef et al. 2021). Endemism follows the same geographic pattern as species richness within the DRC, with higher rates of floristic turnover and species richness per unit area in the eastern and southeastern regions compared to the center of the Congo Basin (Mutke and Barthlott 2005; Sosef et al. 2017). Haut-Katanga is recognized as a “bioquality hotspot,” i.e., with a high proportion of species with a narrow distribution (Marshall et al. 2016).

Many species with narrow distributions in the Albertine Rift and Haut-Katanga have been recorded from a few localities in the neighboring countries of Rwanda and Burundi and Zambia, respectively. The number of such “near-endemics” is certainly very high. This could explain the substantially lower rate of endemism in the DRC compared to Tanzania, the country with the highest number of endemic species in mainland tropical Africa.

Endemics are not randomly distributed across families, ranging from 0% (146 families) to 57% (Hydrostachyaceae). In 100 out of 246 families, at least one endemic species occurs (Suppl. material 2). The ten families with a remarkably high proportion of endemics are listed in Table 5. Most of these species are herbs. Among angiosperms, large families, i.e., those with > 100 species, display highly variable rates of endemism. The following families stand out with a proportion of endemics much higher than average: Commelinaceae (24%: 31/128), Euphorbiaceae (21%: 46/225), Asteraceae (16%: 109/677), and Fabaceae (16%: 194/1,206).

**Table 5.** The 10 families, comprising > 20 species, with the highest rate of endemism in the vascular flora of DRC.

**Family**	**% endemics**	**# endemics**
Santalaceae	44.0%	19
Linderniaceae	33.3%	17
Thymelaeaceae	30.0%	12
Balsaminaceae	27.9%	12
Eriocaulaceae	27.3%	15
Lauraceae	27.3%	9
Iridaceae	25.8%	16
Capparaceae	25.0%	11
Commelinaceae	24.2%	31
Vitaceae	23.4%	25

Genera comprising > 20 species and having substantially larger than average rates of endemism include *Thesium* L. (Santalaceae) (63%: 17/27), *Bothriocline* Oliv. ex Benth. (Asteraceae) (50%: 15/30), *Haumaniastrum* P.A.Duvign. & Plancke (Lamiaceae) (50%: 13/25), *Gladiolus* Tourn. ex L. (Iridaceae) (43%: 12/28), *Cyphostemma* (Planch.) Alston (Vitaceae) (37%: 19/51), *Emilia* Cass. (Asteraceae) (37%: 16/43), *Buchnera* L. (Orobanchaceae) (36%: 15/43), *Beilschmiedia* Nees (Lauraceae) (33%: 9/27), *Humularia* P.A.Duvign. (Fabaceae) (33%: 13/39), *Crotalaria* L. (Fabaceae) (30%: 60/197), *Impatiens* Riv. ex L. (Balsaminaceae) (28%: 12/42), and *Justicia* L. (Acanthaceae) (27%: 15/55). Interestingly, with the exception of *Beilschmiedia* and *Impatiens*, all of these genera are herbs or suffrutices occurring predominantly in Haut-Katanga. The diversity center of many of these genera is in Central Africa (e.g., *Bothriocline*, *Emilia*, *Crotalaria*, *Humularia*, *Haumaniastrum*, *Beilschmiedia*, and *Cyphostemma*), which suggests that the high rates of endemism can be attributed to high diversification rates in the DRC.

By contrast, the following large families, comprising at least 100 spp., have much lower-than-average rates of endemism: Apocynaceae Juss. (5%: 14/285), Cyperaceae Juss. (4.7%: 17/361), and Poaceae (2%: 15/626). The scarcity of narrowly distributed species in these families may be explained by broad ecological niches and occurrence in widespread habitats (Cyperaceae and Poaceae), as well as long-distance dispersal by water birds (Cyperaceae) or fruit-eating mammals and wind (Apocynaceae). Pteridophytes as a group have a very low endemism rate (2.6%: 10/385), which is consistent with their efficient dispersal capacity via airborne spores.

### Introduced species

A total of 461 introduced species have been recorded outside cultivation in the DRC, accounting for 4.5% of the total flora (Table 2). This proportion is similar to that of most other tropical African countries (2–5%, Table 3) but is markedly lower than Chad, Guinea, Mozambique, and São Tomé and Príncipe (all > 10%). However, this result should be interpreted with caution, as recently introduced species are likely to be underrepresented in herbaria.

There are 80 families comprising at least one introduced species (Suppl. material 1) and eight families represented only by introduced species (Basellaceae Raf., Bixaceae Kunth, Bromeliaceae Juss., Cannaceae Juss., Caricaceae Dumort., Heliconiaceae Vines, Moringaceae Martinov, and Tropaeolaceae Juss. ex DC.). Across families, the number of introduced species is significantly correlated with the number of native species (Spearman *r* = 0.50, *p* < 0.00001). Among families with more than 100 species, a lower-than-average proportion of introduced species is found in Orchidaceae Juss. (0.2%), Cyperaceae (0.2%), Acanthaceae Juss. (1.6%), and Rubiaceae (2.2%). Conversely, a higher-than-average proportion of introduced species is observed in Euphorbiaceae (10%), Asteraceae (7%), Poaceae (6.5%), and Fabaceae (6%) (Suppl. material 1). Pteridophytes have very few introduced species (Table 2).

Solanaceae stands out as a remarkable outlier with 23 native vs. 37 introduced species. Other notable families include Brassicaceae Burnett (19 native, 11 introduced) and Amaranthaceae Juss. (61 native, 23 introduced) (Table 6). These three families comprise many domesticated species, which aligns with the global pattern of economic use being a major driver of naturalization (van Kleunen et al. 2020).

**Table 6.** Families, comprising > 20 species, with ≥ 10% introduced species in the DRC.

**Family**	**% introduced species**	**# introduced species**
Solanaceae	62%	37
Brassicaceae	37%	11
Amaranthaceae	27%	23
Plantaginaceae	19%	5
Convolvulaceae	17%	21
Amaryllidaceae	14%	3
Caryophyllaceae	14%	3
Lythraceae	13%	4
Rosaceae	13%	3
Cucurbitaceae	12%	9
Polygonaceae	11%	4
Urticaceae	11%	5
Euphorbiaceae	10%	22

### Growth forms

**Figure 3.**
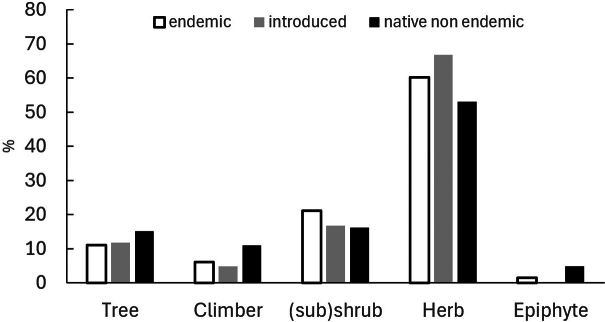
Life form spectrum of the vascular flora of the DRC: comparison of endemics, native non-endemics, and introduced species. The category “epiphyte” includes true epiphytes and epiphytic parasites. The spectrum of life forms is significantly different among the three groups (χ^2^ = 133.38, DF = 8, *p* < 0.000001).

In agreement with broader studies throughout tropical Africa (Droissart et al. 2018), except Gabon (Texier 2022), herbs represent the most frequent life form in the vascular flora of DRC (5,577 species, 54%). This is far higher than the number of (sub)shrubs (1,716), trees (1,492), climbers (1,043), epiphytes (358), and epiphytic parasites (74). There is a significant difference in the life form spectrum across endemics, native non-endemics, and introduced species (χ^2^ = 133.38, DF = 8, *p* < 0.000001) (Fig. 3). Herbs and (sub)shrubs are overrepresented among endemics (herbs: 60% of endemics vs. 54% of native species; (sub)shrubs: 21% vs. 16%). This likely reflects the taxonomic assemblage of endemic species, with herbaceous families being overrepresented. Within the largest family, Fabaceae, there are statistically significant differences in the life form assemblages of endemics and non-endemics (χ^2^ = 19.03, *p* = 0.00027, DF = 3), with trees and climbers being underrepresented among endemics (27% vs. 16.5% and 10% vs. 5.5%, respectively). This aligns well with the fact that endemics are more frequent in savanna and woodland habitats in the southeast of DRC compared to the rainforest.

Herbs are markedly overrepresented among introduced species compared to native species (67% vs. 54%), while climbers and trees are underrepresented (4.8% vs. 10% and 12% vs. 15%, respectively). This aligns with the taxonomic assemblage of introduced species, which is biased toward herbaceous families and short-lived species with high reproductive capacity (e.g., Solanaceae, Amaranthaceae). This pattern has been well documented on a global scale (Dong et al. 2024).

### IUCN conservation status

**Figure 4.**
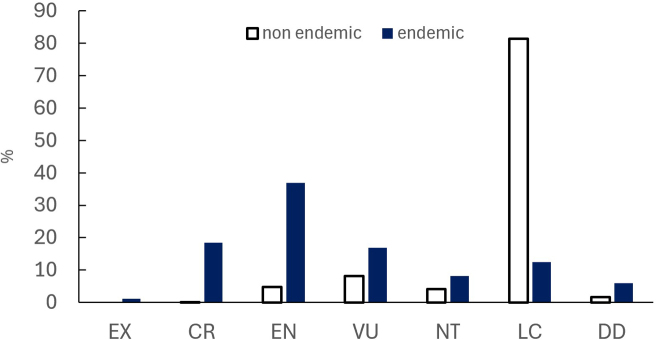
IUCN conservation status of endemic (184) and non-endemic (2,449) species of vascular plants in the DRC. The difference in the proportion of threatened species between endemics and non-endemics is statistically significant (χ^2^ = 442.85, DF = 1, *p* < 0.000001) (EX excluded).

The IUCN conservation status was retrieved for 2,633 species (25.7%). Of these, two species are extinct, and 448 (17%) are assigned to a threat category (CR [Critically Endangered]: 36; EN [Endangered]: 184; VU [Vulnerable]: 228). Of the 1,099 endemic species, 184 (17%) have been evaluated, of which two are extinct (EX) and 133 (73%) are assigned to a threat category (CR: 34; EN: 68; VU: 31). The two extinct species are narrow endemic metallophytes of copper-rich soil in Haut-Katanga, destroyed by mining activities. There is a statistically significant difference in the proportion of threatened species between endemics and non-endemics (χ^2^ = 442.85, DF = 1, *p* < 0.000001) (Fig. 4). This result aligns well with the global pattern of a higher threat of extinction in narrowly distributed species (Knapp 2011). Priority should be given to assessing the conservation status of all endemic species in the DRC for the sake of the country’s biodiversity conservation to build on the Red List of endemic and near-endemic trees (Sosef et al. 2021).

## Conclusion

This study provides the first comprehensive inventory of the vascular flora of the Democratic Republic of the Congo and its threat categories. It documents an exceptionally high level of species richness and endemism, underscoring the country’s critical importance for the conservation of botanical diversity in tropical Africa. It is anticipated that this checklist will serve as a foundation for future floristic and taxonomic research in the country. To complete the inventory of the vascular flora, it will nevertheless be necessary in the future to take into account infraspecific taxa, which could not be considered in the present study.

Substantial knowledge gaps remain and must be addressed through intensified botanical exploration, particularly in regions that are still poorly documented. Taxonomic revision of insufficiently known groups represents an additional priority. A comprehensive assessment of the conservation status of all recorded species is urgently needed (74% of the species have not yet been assessed), prioritizing endemic species, together with updated data on the distribution and spread of introduced alien species. Finally, an improved understanding of the spatial patterns of endemic plant diversity will be essential to inform conservation planning and prioritization efforts.

## Additional information

### Artificial Intelligence (AI) use

The authors accept full responsibility for the content of the manuscript, including the disclosure of any use of AI.

No AI tools were used in the preparation of this manuscript.

### Author ORCIDs

P. Meerts https://orcid.org/0000-0003-4215-027X

P. Barberá https://orcid.org/0000-0002-4110-8419

T.L.P. Couvreur https://orcid.org/0000-0002-8509-6587

R.F. de Almeida https://orcid.org/0000-0002-9562-9287

P. De Block https://orcid.org/0000-0001-7210-7868

M. de la Estrella https://orcid.org/0000-0002-4484-3566

S. Dessein https://orcid.org/0000-0002-3179-4005

H. Engledow https://orcid.org/0000-0002-0779-8006

E. Fischer https://orcid.org/0000-0001-7455-9833

L. Gautier https://orcid.org/0000-0003-4157-3713

A. Haigh https://orcid.org/0000-0003-3435-3501

D.J. Harris https://orcid.org/0000-0002-6801-2484

W. Hetterscheid https://orcid.org/0000-0002-7839-4201

C.C.H. Jongkind https://orcid.org/0000-0002-1491-2030

O. Lachenaud https://orcid.org/0000-0001-8972-4096

I. Larridon https://orcid.org/0000-0003-0285-722X

O. Maurin https://orcid.org/0000-0002-4151-6164

S.J. Mayo https://orcid.org/0000-0002-1969-4984

J. Morel https://orcid.org/0000-0001-7969-2609

I. Mwanga Mwanga https://orcid.org/0000-0003-0203-5795

M.O.O. Pellegrini https://orcid.org/0000-0002-8783-1362

A. Quintanar https://orcid.org/0000-0001-7618-3112

M. Reynders https://orcid.org/0000-0003-1581-6048

E. Robbrecht https://orcid.org/0000-0002-3811-595X

B.I. wa Rusaati https://orcid.org/0000-0001-8092-8682

A.R.G. Simões https://orcid.org/0000-0001-7267-8353

M.S.M. Sosef https://orcid.org/0000-0002-6997-5813

T. Stévart https://orcid.org/0000-0002-6212-0361

R.D. Stone https://orcid.org/0000-0003-2752-7788

A.P. Sukhorukov https://orcid.org/0000-0003-2220-826X

B. Verstraete https://orcid.org/0000-0001-9332-7798

J. Wiland-Szymańska https://orcid.org/0000-0002-1916-7201

M. Xanthos https://orcid.org/0000-0002-5378-8757

F. Zuloaga https://orcid.org/0000-0003-2794-539X

### Data availability

All of the data that support the findings of this study are available in the main text or Supplementary Information.

## Supplementary materials

Supplementary material 1 Checklist of the vascular plants of the Democratic Republic of the Congo Data type xlsx Explanation note For each species, the following information is included: Accepted name; Synonyms; Life form; Endemic status; IUCN status; Voucher specimen (not included for species dealt with in the Flore d’Afrique centrale); reference in the Flore d’Afrique centrale; other literature references; reviewer. See Materials and methods for further explanation. https://binary.pensoft.net/file/1698864This dataset is made available under the Open Database License (http://opendatacommons.org/licenses/odbl/1.0/). The Open Database License (ODbL) is a license agreement intended to allow users to freely share, modify, and use this Dataset while maintaining this same freedom for others, provided that the original source and author(s) are credited. Pierre Meerts, Petra Ballings, Patricia Barberá, Kenneth Bauters, Henk Beentje, Martin Cheek, Thomas L. P. Couvreur, Theo H. J. Damen, Rafael Felipe de Almeida, Petra De Block, Manuel de la Estrella, Steven Dessein, Henry Engledow, Eberhard Fischer, Laurent Gautier, Anna Haigh, David J. Harris, Wilbert Hetterscheid, Carel C. H. Jongkind, Olivier Lachenaud, Isabel Larridon, Alexandra Ley, Olivier Maurin, Simon J. Mayo, Pélagie Mbandu Luzolawo, Chanelle Moafo Tchioffo, Jeremie Morel, Ithe Mwanga Mwanga, Marco O. O. Pellegrini, Alejandro Quintanar, Marc Reynders, Elmar Robbrecht, Butoto Imani wa Rusaati, Ana R. G. Simões, Marc S. M. Sosef, Tariq Stévart, Robert D. Stone, Alexander P. Sukhorukov, Ton van der Zon, Brecht Verstraete, Justyna Wiland-Szymańska, Martin Xanthos, Fernando Zuloaga

Supplementary material 2 The 246 families of vascular plants of the Democratic Republic of the Congo Data type docx Explanation note Summary table of the 246 families of vascular plants of the Democratic Republic of the Congo, with their numbers and proportions of endemic, introduced, and native non-endemic species. https://binary.pensoft.net/file/1698865This dataset is made available under the Open Database License (http://opendatacommons.org/licenses/odbl/1.0/). The Open Database License (ODbL) is a license agreement intended to allow users to freely share, modify, and use this Dataset while maintaining this same freedom for others, provided that the original source and author(s) are credited. Pierre Meerts, Petra Ballings, Patricia Barberá, Kenneth Bauters, Henk Beentje, Martin Cheek, Thomas L. P. Couvreur, Theo H. J. Damen, Rafael Felipe de Almeida, Petra De Block, Manuel de la Estrella, Steven Dessein, Henry Engledow, Eberhard Fischer, Laurent Gautier, Anna Haigh, David J. Harris, Wilbert Hetterscheid, Carel C. H. Jongkind, Olivier Lachenaud, Isabel Larridon, Alexandra Ley, Olivier Maurin, Simon J. Mayo, Pélagie Mbandu Luzolawo, Chanelle Moafo Tchioffo, Jeremie Morel, Ithe Mwanga Mwanga, Marco O. O. Pellegrini, Alejandro Quintanar, Marc Reynders, Elmar Robbrecht, Butoto Imani wa Rusaati, Ana R. G. Simões, Marc S. M. Sosef, Tariq Stévart, Robert D. Stone, Alexander P. Sukhorukov, Ton van der Zon, Brecht Verstraete, Justyna Wiland-Szymańska, Martin Xanthos, Fernando Zuloaga

Supplementary material 3 References Data type docx Explanation note List of literature references cited in the checklist of the vascular plants of the Democratic Republic of the Congo. https://binary.pensoft.net/file/1698866This dataset is made available under the Open Database License (http://opendatacommons.org/licenses/odbl/1.0/). The Open Database License (ODbL) is a license agreement intended to allow users to freely share, modify, and use this Dataset while maintaining this same freedom for others, provided that the original source and author(s) are credited. Pierre Meerts, Petra Ballings, Patricia Barberá, Kenneth Bauters, Henk Beentje, Martin Cheek, Thomas L. P. Couvreur, Theo H. J. Damen, Rafael Felipe de Almeida, Petra De Block, Manuel de la Estrella, Steven Dessein, Henry Engledow, Eberhard Fischer, Laurent Gautier, Anna Haigh, David J. Harris, Wilbert Hetterscheid, Carel C. H. Jongkind, Olivier Lachenaud, Isabel Larridon, Alexandra Ley, Olivier Maurin, Simon J. Mayo, Pélagie Mbandu Luzolawo, Chanelle Moafo Tchioffo, Jeremie Morel, Ithe Mwanga Mwanga, Marco O. O. Pellegrini, Alejandro Quintanar, Marc Reynders, Elmar Robbrecht, Butoto Imani wa Rusaati, Ana R. G. Simões, Marc S. M. Sosef, Tariq Stévart, Robert D. Stone, Alexander P. Sukhorukov, Ton van der Zon, Brecht Verstraete, Justyna Wiland-Szymańska, Martin Xanthos, Fernando Zuloaga
