# Supplementary material for: A checklist of the vascular plants of the Democratic Republic of the Congo
Source: PhytoKeys. 2026 Jun 29;277:1–23. doi: 10.3897/phytokeys.277.193807 (PMC13338719; doi:10.3897/phytokeys.277.193807)
Supplement: Supplementary material 2 — The 246 families of vascular plants of the Democratic Republic of the Congo [file phytokeys-277-001_article-193807__-s002.docx]

**Supplementary material 2**

**Summary table of the 246 families of vascular plants of the Democratic Republic of the Congo, with their numbers and proportions of endemic, introduced, and native non-endemic species**.

| Family | # introduced | # native non-endemic | # endemic | total | % introduced | % endemic |
| --- | --- | --- | --- | --- | --- | --- |
| **Spermatophytes** |  |  |  |  |  |  |
| Acanthaceae | 5 | 281 | 34 | 320 | 1.6% | 10.6% |
| Achariaceae |  | 20 | 3 | 23 | 0.0% | 13.0% |
| Aizoaceae |  | 4 |  | 4 | 0.0% | 0.0% |
| Alismataceae |  | 8 |  | 8 | 0.0% | 0.0% |
| Amaranthaceae | 23 | 57 | 4 | 84 | 27.4% | 4.8% |
| Amaryllidaceae | 3 | 18 |  | 21 | 14.3% | 0.0% |
| Anacardiaceae | 4 | 47 | 10 | 61 | 6.6% | 16.4% |
| Ancistrocladaceae |  | 3 |  | 3 | 0.0% | 0.0% |
| Anisophylleaceae |  | 4 |  | 4 | 0.0% | 0.0% |
| Annonaceae |  | 124 | 10 | 134 | 0.0% | 7.5% |
| Apiaceae | 2 | 49 | 6 | 57 | 3.6% | 10.5% |
| Apocynaceae | 6 | 262 | 12 | 280 | 2.1% | 4.3% |
| Apodanthaceae |  | 1 |  | 1 | 0.0% | 0.0% |
| Aponogetonaceae |  | 5 | 1 | 6 | 0.0% | 16.7% |
| Aquifoliaceae |  | 1 |  | 1 | 0.0% | 0.0% |
| Araceae | 4 | 48 | 7 | 59 | 6.8% | 11.9% |
| Araliaceae |  | 19 | 4 | 23 | 0.0% | 17.4% |
| Arecaceae | 2 | 31 |  | 33 | 6.1% | 0.0% |
| Aristolochiaceae | 4 | 8 |  | 12 | 33.3% | 0.0% |
| Asparagaceae | 2 | 112 | 13 | 127 | 1.6% | 10.2% |
| Asphodelaceae |  | 21 | 5 | 26 | 0.0% | 19.2% |
| Asteraceae | 47 | 523 | 108 | 678 | 6.9% | 15.9% |
| Balanophoraceae |  | 2 |  | 2 | 0.0% | 0.0% |
| Balsaminaceae | 1 | 30 | 12 | 43 | 2.3% | 27.9% |
| Basellaceae | 1 | 0 |  | 1 | 100.0% | 0.0% |
| Begoniaceae |  | 30 | 4 | 34 | 0.0% | 12.1% |
| Bignoniaceae | 2 | 12 |  | 14 | 14.3% | 0.0% |
| Bixaceae | 1 | 2 |  | 3 | 33.3% | 0.0% |
| Boraginaceae | 1 | 25 | 3 | 29 | 3.4% | 10.3% |
| Brassicaceae | 11 | 19 |  | 30 | 36.7% | 0.0% |
| Bromeliaceae | 1 | 0 |  | 1 | 100.0% | 0.0% |
| Burmanniaceae |  | 4 |  | 4 | 0.0% | 0.0% |
| Burseraceae |  | 18 | 3 | 21 | 0.0% | 14.3% |
| Buxaceae |  | 1 | 1 | 2 | 0.0% | 50.0% |
| Cabombaceae |  | 1 |  | 1 | 0.0% | 0.0% |
| Cactaceae | 1 | 1 |  | 2 | 50.0% | 0.0% |
| Calophyllaceae |  | 2 | 1 | 3 | 0.0% | 33.3% |
| Campanulaceae | 1 | 50 | 13 | 64 | 1.6% | 20.3% |
| Canellaceae |  | 1 |  | 1 | 0.0% | 0.0% |
| Cannabaceae | 1 | 9 |  | 10 | 10.0% | 0.0% |
| Cannaceae | 1 | 0 |  | 1 | 100.0% | 0.0% |
| Capparaceae |  | 33 | 11 | 44 | 0.0% | 25.0% |
| Caprifoliaceae | 1 | 6 |  | 7 | 14.3% | 0.0% |
| Cardiopteridaceae |  | 3 |  | 3 | 0.0% | 0.0% |
| Caricaceae | 1 | 0 |  | 1 | 100.0% | 0.0% |
| Caryophyllaceae | 3 | 18 | 1 | 22 | 13.6% | 4.5% |
| Celastraceae |  | 96 | 8 | 104 | 0.0% | 7.7% |
| Ceratophyllaceae |  | 2 |  | 2 | 0.0% | 0.0% |
| Chrysobalanaceae |  | 23 | 1 | 24 | 0.0% | 4.2% |
| Cleomaceae | 2 | 8 | 1 | 11 | 18.2% | 9.1% |
| Clusiaceae |  | 18 | 4 | 22 | 0.0% | 18.2% |
| Colchicaceae |  | 4 |  | 4 | 0.0% | 0.0% |
| Combretaceae | 2 | 75 | 11 | 88 | 2.3% | 12.5% |
| Commelinaceae | 1 | 96 | 31 | 128 | 0.8% | 24.2% |
| Connaraceae |  | 28 | 2 | 30 | 0.0% | 6.7% |
| Convolvulaceae | 21 | 89 | 11 | 121 | 17.4% | 9.1% |
| Corbichoniaceae |  | 1 |  | 1 | 0.0% | 0.0% |
| Cornaceae |  | 2 |  | 2 | 0.0% | 0.0% |
| Costaceae |  | 7 |  | 7 | 0.0% | 0.0% |
| Crassulaceae | 1 | 17 |  | 18 | 5.6% | 0.0% |
| Cucurbitaceae | 9 | 69 |  | 78 | 11.5% | 0.0% |
| Cupressaceae |  | 1 |  | 1 | 0.0% | 0.0% |
| Cyperaceae | 1 | 343 | 17 | 361 | 0.3% | 4.7% |
| Dichapetalaceae |  | 38 | 2 | 40 | 0.0% | 5.0% |
| Dilleniaceae |  | 6 |  | 6 | 0.0% | 0.0% |
| Dioscoreaceae | 1 | 18 | 2 | 21 | 4.8% | 9.5% |
| Dipterocarpaceae |  | 12 | 3 | 15 | 0.0% | 20.0% |
| Droseraceae |  | 8 | 1 | 9 | 0.0% | 11.1% |
| Ebenaceae |  | 38 | 4 | 42 | 0.0% | 9.5% |
| Elatinaceae |  | 1 |  | 1 | 0.0% | 0.0% |
| Ericaceae |  | 9 |  | 9 | 0.0% | 0.0% |
| Eriocaulaceae |  | 40 | 15 | 55 | 0.0% | 27.3% |
| Erythroxylaceae |  | 5 | 2 | 7 | 0.0% | 28.6% |
| Euphorbiaceae | 22 | 159 | 44 | 225 | 9.8% | 19.6% |
| Fabaceae | 71 | 953 | 182 | 1206 | 5.9% | 15.1% |
| Flagellariaceae |  | 1 |  | 1 | 0.0% | 0.0% |
| Francoaceae |  | 3 |  | 3 | 0.0% | 0.0% |
| Gelsemiaceae |  | 3 |  | 3 | 0.0% | 0.0% |
| Gentianaceae |  | 44 | 8 | 52 | 0.0% | 15.4% |
| Geraniaceae |  | 12 |  | 12 | 0.0% | 0.0% |
| Gesneriaceae |  | 6 | 5 | 11 | 0.0% | 45.5% |
| Gisekiaceae |  | 2 | 1 | 3 | 0.0% | 33.3% |
| Gnetaceae |  | 3 |  | 3 | 0.0% | 0.0% |
| Goodeniaceae |  | 1 |  | 1 | 0.0% | 0.0% |
| Gunneraceae |  | 1 |  | 1 | 0.0% | 0.0% |
| Haloragaceae |  | 1 |  | 1 | 0.0% | 0.0% |
| Hamamelidaceae |  | 1 |  | 1 | 0.0% | 0.0% |
| Heliconiaceae |  | 1 |  | 1 | 0.0% | 0.0% |
| Hernandiaceae |  | 2 |  | 2 | 0.0% | 0.0% |
| Huaceae |  | 3 |  | 3 | 0.0% | 0.0% |
| Humiriaceae |  | 1 |  | 1 | 0.0% | 0.0% |
| Hydrocharitaceae |  | 21 |  | 21 | 0.0% | 0.0% |
| Hydroleaceae |  | 1 |  | 1 | 0.0% | 0.0% |
| Hydrostachyaceae |  | 3 | 4 | 7 | 0.0% | 57.1% |
| Hypericaceae |  | 20 |  | 20 | 0.0% | 0.0% |
| Hypoxidaceae |  | 14 | 6 | 20 | 0.0% | 30.0% |
| Icacinaceae |  | 27 | 2 | 29 | 0.0% | 6.9% |
| Iridaceae | 2 | 44 | 16 | 62 | 3.2% | 25.8% |
| Irvingiaceae |  | 10 |  | 10 | 0.0% | 0.0% |
| Iteaceae |  | 1 |  | 1 | 0.0% | 0.0% |
| Ixonanthaceae |  | 3 |  | 3 | 0.0% | 0.0% |
| Juncaceae |  | 6 |  | 6 | 0.0% | 0.0% |
| Juncaginaceae |  | 2 |  | 2 | 0.0% | 0.0% |
| Kirkiaceae |  | 1 |  | 1 | 0.0% | 0.0% |
| Lamiaceae | 10 | 249 | 47 | 306 | 3.3% | 15.8% |
| Lauraceae |  | 24 | 9 | 33 | 0.0% | 27.3% |
| Lecythidaceae |  | 10 | 2 | 12 | 0.0% | 16.7% |
| Lentibulariaceae |  | 31 |  | 31 | 0.0% | 0.0% |
| Lepidobotryaceae |  | 1 |  | 1 | 0.0% | 0.0% |
| Liliaceae | 1 | 0 |  | 1 | 100.0% | 0.0% |
| Limeaceae |  | 1 | 1 | 2 | 0.0% | 50.0% |
| Linaceae |  | 11 |  | 11 | 0.0% | 0.0% |
| Linderniaceae | 1 | 35 | 17 | 53 | 1.9% | 33.3% |
| Loganiaceae | 1 | 42 |  | 43 | 2.3% | 0.0% |
| Loranthaceae |  | 62 | 2 | 64 | 0.0% | 3.1% |
| Lythraceae | 4 | 26 | 2 | 32 | 12.5% | 6.3% |
| Malpighiaceae |  | 15 |  | 15 | 0.0% | 0.0% |
| Malvaceae | 13 | 194 | 35 | 242 | 5.4% | 14.5% |
| Marantaceae |  | 24 |  | 24 | 0.0% | 0.0% |
| Mayacaceae |  | 1 |  | 1 | 0.0% | 0.0% |
| Melastomataceae | 1 | 88 | 15 | 104 | 1.0% | 14.4% |
| Meliaceae | 4 | 49 |  | 53 | 7.5% | 0.0% |
| Menispermaceae |  | 41 | 2 | 43 | 0.0% | 4.7% |
| Menyanthaceae |  | 5 |  | 5 | 0.0% | 0.0% |
| Metteniusaceae |  | 4 |  | 4 | 0.0% | 0.0% |
| Molluginaceae | 1 | 9 |  | 10 | 10.0% | 0.0% |
| Monimiaceae |  | 1 |  | 1 | 0.0% | 0.0% |
| Montiaceae |  | 1 |  | 1 | 0.0% | 0.0% |
| Moraceae | 2 | 85 | 5 | 92 | 2.2% | 5.4% |
| Moringaceae | 1 | 0 |  | 1 | 100.0% | 0.0% |
| Musaceae | 1 | 3 |  | 4 | 25.0% | 0.0% |
| Myricaceae |  | 6 |  | 6 | 0.0% | 0.0% |
| Myristicaceae |  | 5 |  | 5 | 0.0% | 0.0% |
| Myrothamnaceae |  | 1 |  | 1 | 0.0% | 0.0% |
| Myrtaceae | 2 | 24 | 1 | 27 | 7.4% | 3.7% |
| Nyctaginaceae | 3 | 5 |  | 8 | 37.5% | 0.0% |
| Nymphaeaceae |  | 6 |  | 6 | 0.0% | 0.0% |
| Ochnaceae |  | 46 | 4 | 50 | 0.0% | 8.0% |
| Olacaceae |  | 22 | 3 | 25 | 0.0% | 12.0% |
| Oleaceae |  | 17 | 1 | 18 | 0.0% | 5.6% |
| Onagraceae | 3 | 12 |  | 15 | 20.0% | 0.0% |
| Opiliaceae |  | 5 |  | 5 | 0.0% | 0.0% |
| Orchidaceae | 1 | 523 | 48 | 572 | 0.2% | 8.4% |
| Orobanchaceae |  | 76 | 20 | 96 | 0.0% | 20.8% |
| Oxalidaceae | 5 | 13 |  | 18 | 27.8% | 0.0% |
| Pandaceae |  | 4 | 2 | 6 | 0.0% | 33.3% |
| Pandanaceae |  | 3 |  | 3 | 0.0% | 0.0% |
| Papaveraceae | 2 | 2 |  | 4 | 50.0% | 0.0% |
| Passifloraceae | 5 | 42 | 10 | 57 | 8.8% | 17.5% |
| Pedaliaceae | 1 | 7 |  | 8 | 12.5% | 0.0% |
| Penaeaceae |  | 3 |  | 3 | 0.0% | 0.0% |
| Pentadiplandraceae |  | 1 |  | 1 | 0.0% | 0.0% |
| Pentaphylacaceae |  | 1 |  | 1 | 0.0% | 0.0% |
| Peraceae |  | 5 | 1 | 6 | 0.0% | 16.7% |
| Petiveriaceae | 2 | 1 |  | 3 | 66.7% | 0.0% |
| Phrymaceae |  | 1 |  | 1 | 0.0% | 0.0% |
| Phyllanthaceae | 3 | 112 | 13 | 128 | 2.3% | 10.2% |
| Phytolaccaceae | 2 | 1 |  | 3 | 66.7% | 0.0% |
| Picrodendraceae |  | 1 | 1 | 2 | 0.0% | 50.0% |
| Piperaceae | 2 | 11 | 1 | 14 | 14.3% | 7.1% |
| Pittosporaceae |  | 4 |  | 4 | 0.0% | 0.0% |
| Plantaginaceae | 5 | 21 |  | 26 | 19.2% | 0.0% |
| Plumbaginaceae |  | 2 | 1 | 3 | 0.0% | 33.3% |
| Poaceae | 41 | 570 | 15 | 626 | 6.5% | 2.4% |
| Podocarpaceae |  | 5 |  | 5 | 0.0% | 0.0% |
| Podostemaceae |  | 10 | 3 | 13 | 0.0% | 23.1% |
| Polygalaceae |  | 50 | 2 | 52 | 0.0% | 3.8% |
| Polygonaceae | 4 | 28 | 4 | 36 | 11.1% | 11.1% |
| Pontederiaceae | 1 | 3 |  | 4 | 25.0% | 0.0% |
| Portulacaceae | 1 | 5 |  | 6 | 16.7% | 0.0% |
| Potamogetonaceae |  | 7 |  | 7 | 0.0% | 0.0% |
| Primulaceae |  | 32 | 4 | 36 | 0.0% | 11.1% |
| Proteaceae |  | 19 | 1 | 20 | 0.0% | 5.0% |
| Psilotaceae |  | 1 |  | 1 | 0.0% | 0.0% |
| Putranjivaceae |  | 26 |  | 26 | 0.0% | 0.0% |
| Ranunculaceae |  | 22 | 3 | 25 | 0.0% | 12.0% |
| Restionaceae |  | 1 |  | 1 | 0.0% | 0.0% |
| Rhamnaceae |  | 19 | 2 | 21 | 0.0% | 9.5% |
| Rhizophoraceae |  | 14 |  | 14 | 0.0% | 0.0% |
| Rosaceae | 3 | 18 | 3 | 24 | 12.5% | 12.5% |
| Rubiaceae | 12 | 625 | 57 | 694 | 1.7% | 8.2% |
| Ruppiaceae |  | 1 |  | 1 | 0.0% | 0.0% |
| Rutaceae | 1 | 33 | 3 | 37 | 2.8% | 8.1% |
| Salicaceae |  | 28 | 4 | 32 | 0.0% | 12.5% |
| Salvadoraceae |  | 1 |  | 1 | 0.0% | 0.0% |
| Santalaceae |  | 24 | 19 | 43 | 0.0% | 44.2% |
| Sapindaceae | 1 | 64 | 12 | 77 | 1.3% | 15.6% |
| Sapotaceae |  | 65 | 2 | 67 | 0.0% | 3.0% |
| Scrophulariaceae | 1 | 10 | 1 | 12 | 8.3% | 8.3% |
| Simaroubaceae | 1 | 7 | 2 | 10 | 10.0% | 20.0% |
| Sladeniaceae |  | 1 |  | 1 | 0.0% | 0.0% |
| Smilacaceae |  | 2 |  | 2 | 0.0% | 0.0% |
| Solanaceae | 37 | 23 |  | 60 | 61.7% | 0.0% |
| Sphenocleaceae |  | 1 |  | 1 | 0.0% | 0.0% |
| Stemonuraceae |  | 1 |  | 1 | 0.0% | 0.0% |
| Stilbaceae |  | 4 |  | 4 | 0.0% | 0.0% |
| Talinaceae | 2 | 1 |  | 3 | 66.7% | 0.0% |
| Tecophilaeaceae |  | 3 |  | 3 | 0.0% | 0.0% |
| Thomandersiaceae |  | 5 |  | 5 | 0.0% | 0.0% |
| Thymelaeaceae |  | 27 | 12 | 39 | 0.0% | 30.8% |
| Tropaeolaceae | 1 | 0 |  | 1 | 100.0% | 0.0% |
| Typhaceae |  | 3 |  | 3 | 0.0% | 0.0% |
| Ulmaceae |  | 1 |  | 1 | 0.0% | 0.0% |
| Urticaceae | 5 | 42 |  | 47 | 10.6% | 0.0% |
| Velloziaceae |  | 8 |  | 8 | 0.0% | 0.0% |
| Verbenaceae | 8 | 12 |  | 20 | 40.0% | 0.0% |
| Viburnaceae | 1 | 0 |  | 1 | 100.0% | 0.0% |
| Violaceae |  | 35 | 7 | 42 | 0.0% | 16.7% |
| Vitaceae |  | 82 | 25 | 107 | 0.0% | 23.4% |
| Vochysiaceae |  | 1 |  | 1 | 0.0% | 0.0% |
| Xyridaceae |  | 32 | 6 | 38 | 0.0% | 15.8% |
| Zamiaceae |  | 6 | 1 | 7 | 0.0% | 14.3% |
| Zingiberaceae |  | 38 | 8 | 46 | 0.0% | 17.4% |
| Zygophyllaceae |  | 3 |  | 3 | 0.0% | 0.0% |
|  |  |  |  |  |  |  |
| **Lycophytes and Monilophytes** |  |  |  |  |  |  |
| Anemiaceae |  | 5 |  | 5 | 0.0% | 0.0% |
| Arthropteridaceae |  | 3 |  | 3 | 0.0% | 0.0% |
| Aspleniaceae |  | 59 |  | 59 | 0.0% | 0.0% |
| Athyriaceae |  | 12 |  | 12 | 0.0% | 0.0% |
| Blechnaceae |  | 4 |  | 4 | 0.0% | 0.0% |
| Cyatheaceae |  | 6 | 1 | 7 | 0.0% | 14.3% |
| Cystopteridaceae |  | 1 |  | 1 | 0.0% | 0.0% |
| Davalliaceae |  | 2 |  | 2 | 0.0% | 0.0% |
| Dennstaedtiaceae |  | 13 | 1 | 14 | 0.0% | 7.1% |
| Didymochlaenaceae |  | 2 |  | 2 | 0.0% | 0.0% |
| Dryopteridaceae |  | 38 |  | 38 | 0.0% | 0.0% |
| Equisetaceae |  | 1 |  | 1 | 0.0% | 0.0% |
| Gleicheniaceae |  | 4 |  | 4 | 0.0% | 0.0% |
| Hymenophyllaceae |  | 28 |  | 28 | 0.0% | 0.0% |
| Isoetaceae |  | 0 | 1 | 1 | 0.0% | 100.0% |
| Lindsaeaceae |  | 2 |  | 2 | 0.0% | 0.0% |
| Lomariopsidaceae |  | 6 |  | 6 | 0.0% | 0.0% |
| Lonchitidaceae |  | 1 |  | 1 | 0.0% | 0.0% |
| Lycopodiaceae |  | 13 | 1 | 14 | 0.0% | 7.1% |
| Lygodiaceae |  | 2 |  | 2 | 0.0% | 0.0% |
| Marattiaceae |  | 1 |  | 1 | 0.0% | 0.0% |
| Marsileaceae |  | 2 |  | 2 | 0.0% | 0.0% |
| Nephrolepidaceae |  | 3 |  | 3 | 0.0% | 0.0% |
| Oleandraceae |  | 1 |  | 1 | 0.0% | 0.0% |
| Ophioglossaceae |  | 10 |  | 10 | 0.0% | 0.0% |
| Osmundaceae |  | 1 |  | 1 | 0.0% | 0.0% |
| Polypodiaceae |  | 21 |  | 21 | 0.0% | 0.0% |
| Psilotaceae |  | 1 |  | 1 | 0.0% | 0.0% |
| Pteridaceae | 1 | 74 | 2 | 77 | 1.3% | 2.6% |
| Salviniaceae | 2 | 3 |  | 5 | 40.0% | 0.0% |
| Selaginellaceae |  | 19 | 4 | 23 | 0.0% | 17.4% |
| Tectariaceae |  | 16 |  | 16 | 0.0% | 0.0% |
| Thelypteridaceae |  | 17 | 1 | 18 | 0.0% | 5.6% |
|  |  |  |  |  |  |  |
