# Supplementary material for: A checklist of the vascular plants of the Democratic Republic of the Congo
Source: PhytoKeys. 2026 Jun 29;277:1–23. doi: 10.3897/phytokeys.277.193807 (PMC13338719; doi:10.3897/phytokeys.277.193807)
Supplement: Supplementary material 3 — References [file phytokeys-277-001_article-193807__-s003.docx]

**Supplementary material 3**

**List of literature references cited in the checklist of the vascular plants of the Democratic Republic of the Congo.**

**1. Flore d’Afrique centrale** (formerly Flore du Congo belge et du Ruanda-Urundi, Flore du Congo, du Rwanda et du Burundi)

All fascicles published before 2023 are available at:
<https://www.plantentuinmeise.be/en/fac-family-list-9fbm>

Fascicles published after 2022:

Bedigian D (2024) Pedaliaceae. In: Sosef M.S.M. (ed.), Flore d’Afrique centrale, nouvelle série, Spermatophyta. Meise, Jardin botanique de Meise. 31 p.

Beentje H, Ballings P (in ed.) Aspleniaceae. In: Hennequin S (Ed.) Flore d’Afrique centrale, nouvelle série, Pteridophyta. Meise, Jardin botanique de Meise.

Fischer E (2025) Orobanchaceae. In: Sosef M.S.M. (ed.), Flore d’Afrique centrale, nouvelle série, Spermatophyta. Meise, Jardin botanique de Meise. 226 p.

Ilunga wa Ilunga E & Meerts P (2024) Verbenaceae. In: Sosef MSM (Ed.), Flore d’Afrique centrale, nouvelle série, Spermatophyta. Meise, Jardin botanique de Meise. 60 p.

Meerts P & Paton A. (2025) Labiatae, sous-familles VI. Nepetoideae et VII. Tectonoideae. In: Sosef MSM (ed.), Flore d’Afrique centrale, nouvelle série, Spermatophyta. Meise, Jardin botanique de Meise. 472 p.

Meerts P (2023) Labiatae, Sous-famille III. Ajugoideae. In: Sosef MSM (Ed.), Flore d’Afrique centrale, nouvelle série, Spermatophyta. Meise, Jardin botanique de Meise. 128 p.

Meerts P (2024) Araliaceae, sous-famille II. Hydrocotyloideae. In: Sosef M.S.M. (Ed.), Flore d’Afrique centrale, nouvelle série. Spermatophyta. Meise, Jardin botanique de Meise. 18 p.

Meerts P (2025) Petiveriaceae. In: Sosef MSM (Ed.), Flore d’Afrique centrale, nouvelle série, Spermatophyta. Meise, Jardin botanique de Meise. 17 p.

Moafo C, Reynders M (2025) Cyperaceae, Tribu XI. Eleochariteae. In: Sosef MSM (Ed.), Flore d’Afrique centrale, nouvelle série, Spermatophyta. Meise, Jardin botanique de Meise. 34 p.

Ntore S, Lachenaud O, Sonké B (2023) Rubiaceae – Tribu XII. Sherbournieae. In: Sosef MSM (Ed.), Flore d'Afrique centrale, nouvelle série. Spermatophyta. Meise, Jardin Botanique de Meise. 76 p.

Oulo M, Guang-Wan Hu & Sosef MSM (2023) Gramineae – Tribu XXX. Cynodonteae. In: Sosef MSM (Ed.), Flore d'Afrique centrale, nouvelle série. Spermatophyta. Jardin Botanique de Meise, 139 p.

Phillips S, Kimpouni V (2024) Eriocaulaceae. In: Sosef MSM (Ed.), Flore d’Afrique centrale, nouvelle série, Spermatophyta. Meise, Jardin botanique de Meise. 119 p.

Robbrecht E, Mwanga Mwanga IJ-C, Stoffelen P (2024) Rubiaceae, Tribu VIII. Coffeeae. In: Sosef MSM (Ed.) Flore d’Afrique centrale, nouvelle série, Spermatophyta. Meise, Jardin botanique de Meise. 184 p.

Sosef MSM, Robert O, Vande Kerkhof KL (2025) Gramineae, Tribu XXIII. Arundineae à XXVI. Danthonieae. In: Sosef MSM (Ed.), Flore d’Afrique centrale, nouvelle série, Spermatophyta. Meise, Jardin botanique de Meise. 44 p.

Thery P, Mesterházy A (2024) Cyperaceae, Tribu XII. Fuireneae. In: Sosef M.S.M. (ed.), Flore d’Afrique centrale, nouvelle série, Spermatophyta. Meise, Jardin botanique de Meise. 60 p.

**Other references**

Alston AHG (1956) New African ferns. Boletim da Sociedade Broteriana, Sér. 2, 30: 5–27.

Baldwin I, Cheek M (2019) *Cola elegans*. The IUCN Red List of Threatened Species 2019: e.T111392197A111449277. https://dx.doi.org/10.2305/IUCN.UK.2019-1.RLTS.T111392197A111449277.en. Accessed on 08 December 2025.

Barberá P, Velayos M, Aedo C (2014) Taxonomic revision of *Grossera* (Crotonoideae, Euphorbiaceae): A central African genus. Systematic Botany 39(2): 490–509. DOI: 10.1600/036364414X680861

Beentje H, Lachenaud O (2020) The inclusion of *Akeassia* in *Grangea* (Asteraceae) and description of a new species from Gabon: *Grangea ogoouensis*. Candollea 75(2): 311–319. DOI**:** 10.15553/c2020v752a9

Breteler F (2018) Revision of the African genus *Crotonogyne* (Euphorbiaceae). Plant Ecology and Evolution 151 (3): 352–379. DOI**:** 10.5091/plecevo.2018.1507

Cardiel JM, Montero-Muñoz I, Gamarra R, Ortúñez E, Muñoz-Rodríguez P (2025) A nomenclatural review of *Acalypha* L. (Euphorbiaceae) of mainland Africa. Taxon 74(5): 1217–1247.

Chevalier A (1908) Novitates floræ africanæ. Bulletin de la Société Botanique de France, 55 (Mém. 8b): 31–109.

De Block P (1998) The African species of *Ixora* (Rubiaceae-Pavetteae). Opera Botanica Belgica 9: 1–218.

de Wilde WJJO (1971) A monograph of the genus *Adenia* Forsk. (Passifloraceae). Mededelingen Landbouwhogeschool Wageningen 71(18).

De Wildeman É (1924) Rubiaceae. In: Plantae Bequaertianae. Études sur les récoltes botaniques du Dr. J. Bequaert, chargé de missions au Congo belge (1913–1915). Volume 2, Part 3. A. Buyens, Gand, 309–482. <https://www.biodiversitylibrary.org/page/62154072>

De Wildeman É, Durand T (1899) Rubiaceae. In: De Wildeman É, Durand T (Eds) Contributions à la flore du Congo. Annales du Musée du Congo (Belge), Botanique, Série II, Tome 1, Fascicule 1. Charles Vande weghe, Bruxelles, 27–30. <https://bibdigital.rjb.csic.es/idurl/1/15815>

Degreef J (2006) Revision of continental African *Tarenna* (Rubiaceae-Pavetteae). Opera Botanica Belgica 14: 1–150.

Descourvières P et al. (2018) A new genus of angraecoid orchids (Orchidaceae: Angraecinae) with highly distinctive pollinaria morphology, including three new species from tropical West and Central Africa. Phytotaxa 373, 99–120, <https://doi.org/10.11646/phytotaxa.373.2.1>

Estrella M de la, Aedo C, Mackinder B & Velayos M (2010) Taxonomic Revision of Daniellia (Leguminosae: Caesalpinioideae). Systematic Botany, 35(2): 296–324. <https://doi.org/10.1600/036364410791638414>

Farminhão JN, D'haijère T, Droissart V, Isonga LD, Dong L, Verlynde S, ... & Stévart T (2020) An Elegy to *Rangaeris*, Including a Description of Two New Genera in the *Cyrtorchis–Tridactyle* Clade (Orchidaceae, Angraecinae) 1. Annals of the Missouri Botanical Garden 105(3): 300–322. https://doi.org/10.3417/2020472

Fischer E, Beentje HJ, Kabuye C, Kalema J, Kayombo C, Luke WRQ, Nshutiyayesu S, Ntore S (2019) *Microcoelia nyungwensis*. The IUCN Red List of Threatened Species 2019: e.T16574410A16574652. https://dx.doi.org/10.2305/IUCN.UK.2019-3.RLTS.T16574410A16574652.en. Accessed on 07 December 2025.

Fischer E, Lebel J-P, Delepierre G, Stévart G, Farminhão J (2024b) *Liparis killmanniae*, a new species of *Liparis* from the Democratic Republic of Congo, Rwanda, Burundi and Uganda and the identity of Liparis deistelii Schltr. Die Orchidee 10(01): 12.

Fischer E, Lobin W (2023) Synoptic Revision of Aspleniaceae (*Asplenium, Hymenasplenium*) of Rwanda. Phytotaxa 608 (1): 1–65. DOI: 10.11646/phytotaxa.608.1.1

Fischer E, Nsanzurwimo A, Dumbo B, Richter R, Vande weghe JP (2024a) An updated checklist of vascular plants (Lycophytes, Ferns, Gymnosperms, Angiosperms) from Nyungwe National Park (incl. Cyamudongo Forest), Rwanda. Phytotaxa 673(1): 1‒113. DOI: 10.11646/phytotaxa.673.1.1.

Harris DJ, Barberá P, Nguema D, Quintanar A (2021) Putranjivaceae Endl. In: Sosef MSM, Florence J, Bourobou H, Bissiengou P (Eds). Flore du Gabon 57: 108‒160. Weikersheim: Margraf Publishers.

Hoekstra PH, Wieringa JJ, Maas PJM, Chatrou L (2021) Revision of the African species of *Monanthotaxis* (Annonaceae). Blumea 66(2): 107–221. <https://doi.org/10.3767/blumea.2021.66.02.01>

Huygh W, Goetghebeur P, Browning J, Larridon I (in press) A global revision of *Cyperus* section *Kyllinga* (*Cyperus* subgenus *Cyperus*, Cypereae, Cyperaceae). Kew Bulletin.

Jacques-Félix H (1974) Le genre *Dicellandra* Hook.f. (Mélastomacées). Adansonia, ser. 2, 14: 77–98.

Jeffrey C (1997) What is *Emilia coccinea* (Sims) G.Don (Compositae)? A revision of the large-headed *Emilia* species of Africa. Kew Bulletin 52(1): 205‒212. DOI**:** 10.2307/4117843

Jongkind CCH (2011) *Vernonia excelsa*, a new liana from Central Africa, and notes on related species. Kew Bulletin 66: 179–182. DOI**:** 10.1007/s12225-011-9275-7

Jongkind CCH (2021) A review of *Combretum falcatum* (Welw. ex Hiern) Jongkind (Combretaceae) and related species from Africa, including *Combretum baldwinii* Jongkind, sp. nov., from Nigeria. Adansonia, sér. 3, 43 (22): 241–249. https://doi.org/10.5252/adansonia2021v43a22. http://adansonia.com/43/22

Jongkind CCH, Lachenaud O (2022) Novelties in African Apocynaceae. Candollea 77: 17–51.

Kalanda K, Lisowski S (1995) Le genre *Vernonia* (Asteraceae) dans la flore d'Afrique centrale (Zaire, Rwanda, Burundi). Fragmenta Floristica et Geobotanica 40 (2): 547–717.

Kalanda K (1986) Contribution à l’étude des Vernonieae (Asteraceae) d’Afrique centrale. Bulletin du Jardin botanique National de Belgique 56: 383–388.

Kamau PW (2012) Systematic revision of *Pteris* in tropical Africa and ecology of ferns and Lycophytes in lowland tropical rainforests. PhD Thesis, University of Koblenz-Landau.

Kenfack et al. (2015) The Tropical African Genus *Crotonogynopsis* (Euphorbiaceae) with two new species. Novon 24: 246–255. DOI: 10.3417/2015005

Kornas A et al. (2000) Pteridophytes of upper Katanga (DRC). Botanical Papers (Prace Botaniczne) 35.

Lachenaud O (2019) Révision du genre *Psychotria* (Rubiaceae) en Afrique Occidentale et Centrale. Opera Botanica Belgica 17: 1–909.

Lachenaud O, Ruiz de Diego P, Riina R & Barberá P (2026) *Ewangoa*, a new threatened African genus of Euphorbiaceae-Acalyphoideae. Taxon 75(2): e70094.

Lachenaud O, van der Maesen LJG (2016) Notes on African *Dalbergia* (Leguminosae – Papilionoideae) with the description of two new species from Atlantic Central Africa. Symbolae Botanicae Upsalienses 38: 167–194.

Larridon I, Reynders M, Huygh W, Bauters K, Vrijdaghs A, Leroux O, Muasya AM, Simpson DA, Goetghebeur P (2011) Taxonomic changes in C_3_ *Cyperus* (Cyperaceae) supported by molecular data, morphology, embryography, ontogeny and anatomy. Plant Ecology and Evolution 144(3): 327–356. 10.5091/plecevo.2011.653

Lisowski S (1996a) Une espéce nouvelle du genre *Senecio* (Asteraceae) de l'Afrique centrale. Fragmenta Floristica et Geobotanica 41 (1): 484–485.

Lisowski S (1996b) Trois espèces nouvelles du genre *Bothriocline* (Asteraceae, Vernonieae) du Zaire. Fragmenta Floristica et Geobotanica 41 (1): 487–489.

Lisowski S (1991) Les Asteraceae dans la flore d’Afrique centrale (excl. Cichorieae, Inuleae et et Vernonieae). Fragmenta Floristica et Geobotanica 36(1) Suppl. 1(1): 1–249; 1(2): 251–627.

Lisowski S (1992) Les Vernonieae (Asteraceae) dans la flore d'Afrique centrale (excl. le genre Vernonia). Fragmenta Floristica et Geobotanica 37(2): 275–369.

Macedo A, Droissart V, Janeček Š, Klomberg Y, Trovó M, Stévart T, Farminhão J (2026) Two new species of *Rhipidoglossum* (Orchidaceae, Angraecinae) from Central Africa, probably pollinated by settling moths. PhytoKeys 274: 1–30. https://doi.org/10.3897/phytokeys.274.184429

MacLeish NFF (1984) Eight New Combinations in *Vernonia* (Compositae: Vernonieae). Systematic Botany 9: 133–136. DOI**:** 10.2307/2418615

Mangambu Mokoso et al. (2013) Espèces nouvellement signalées pour la flore ptéridologique de la République Démocratique du Congo. International Journal of Biological and Chemical Sciences 7(1): 107–124.

Mestre Serra E, Puglisi C, Linan AG, Meeprom N, Rakouth HN, Schmidt HH, Lowry II PP (2025) A taxonomic revision of the continental African material previously included in *Diospyros ferrea* (Ebenaceae). Plant Ecology and Evolution 158(1): 82–99. <https://doi.org/10.5091/plecevo.140561>

Ohoto Omana Losokola E (1980) Morphologie comparée des espèces du genre *Eragrostis* Wolf (Gramineae) du Zaïre. Thèse de doctorat, KULeuven.

Pichi Sermolli REG (1973) Fragmenta Pteridologiae III. Webbia 27(2): 398‒459.

Pichi Sermolli REG (1983a) Fragmenta Pteridologiae VIII. Webbia 37(1): 111–140.

Pichi Sermolli REG (1983b) A contribution to the knowledge of the Pteridophyta of Rwanda, Burundi and Kivu (Zaire). I. Bulletin du Jardin botanique national de Belgique 53: 177–284.

Pichi Sermolli REG (1985) A contribution to the knowledge of the Pteridophyta of Rwanda, Burundi and Kivu (Zaire). II. Bulletin du Jardin botanique national de Belgique 55: 123–206.

Prance GT, Jongkind CCH (2016) A revision of African Lecythidaceae. Kew Bulletin 70: 1–68. DOI**:** 10.1007/S12225-015-9611-1

Quintanar A, Barberá P, Elliott A, Harris DJ (2025a) The Putranjivaceae of the world: a cross-referenced checklist of the genera and species. Edinburgh Journal of Botany 82: 1–146. https://doi.org/10.24823/EJB.2025.2058

Quintanar A, Cheek MR, Barbera P, Harris DJ (2025b) Lost in the swamp: *Drypetes corrugata*, a threatened new species from Central Africa, and its two sister species, *D. chevalieri* and *D. tessmanniana* (Putranjivaceae). Candollea 80: 197–211.

Robbrecht E, Rohrhofer U, Puff C (1993) A survey of *Bertiera* (Rubiaceae), including a discussion of its taxonomic position. Opera Botanica Belgica 6: 101–141.

Schwartsburd PB et al. (2016) Additions to the taxonomy of the *Hypolepis rugosula* complex (Dennstaedtiaceae) in Africa: corrections, two new subspecies and new distribution maps. Folia Geobotanica 51: 373–381. DOI: 10.1007/s12224-016-9261-3

Simo-Droissart M, Stévart T, Pollard BJ, Droissart V (2021) *Bulbophyllum teretifolium*. The IUCN Red List of Threatened Species 2021: e.T87584757A87739987. https://dx.doi.org/10.2305/IUCN.UK.2021-3.RLTS.T87584757A87739987.en. Accessed on 09 December 2025.

Taedoumg HE (2020) Taxonomie du genre *Craterispernum* Benth. (Gentianales - Rubiaceae) en Afrique continentale. Abc Taxa 20: 1–169.

Thomson JA et al. (2005) The taxonomic status and relationships of bracken ferns (*Pteridium*: Dennstaedtiaceae) from sub-Saharan Africa. Botanical Journal of the Linnean Society 148: 311–321. DOI**:** 10.1111/j.1095-8339.2005.00407.x

Timberlake JR (2023) *Euphorbia triangolensis*. The IUCN Red List of Threatened Species 2023: e.T209832512A209944330. https://dx.doi.org/10.2305/IUCN.UK.2023-1.RLTS.T209832512A209944330.en. Accessed on 07 December 2025.

Viennot-Bourgin G (Ed.) (1960) Rapports du sol et de la végétation. Premier colloque de la Société botanique de France, Paris, 13 Juin 1959. Paris, Masson.

Wheatcroft H (2025) *Euphorbia depauperata*. The IUCN Red List of Threatened Species 2025: e.T136532102A234673789. https://dx.doi.org/10.2305/IUCN.UK.2025-2.RLTS.T136532102A234673789.en. Accessed on 09 December 2025.

Zemagho MLA (2016) Phylogeny and taxonomy of continental African *Sabicea* (Rubiaceae). Doctoral dissertation. University of Bayreuth, Germany. <https://epub.uni-bayreuth.de/id/eprint/2803/>
